# Flux Connections Between Gluconate Pathway, Glycolysis, and Pentose–Phosphate Pathway During Carbohydrate Metabolism in *Bacillus megaterium* QM B1551

**DOI:** 10.3389/fmicb.2018.02789

**Published:** 2018-11-21

**Authors:** Julie A. Wushensky, Tracy Youngster, Caroll M. Mendonca, Ludmilla Aristilde

**Affiliations:** ^1^Department of Biological and Environmental Engineering, College of Agriculture and Life Sciences, Cornell University, Ithaca, NY, United States; ^2^Soil and Crop Sciences Section, School of Integrative Plant Science, College of Agriculture and Life Sciences, Cornell University, Ithaca, NY, United States

**Keywords:** metabolomics of carbohydrate catabolism, glucose and fructose assimilation, gluconate uptake and metabolism, fructose metabolism, oxidative pentose–phosphate pathway, *Bacillus megaterium*

## Abstract

*Bacillus megaterium* is a bacterium of great importance as a plant-beneficial bacterium in agricultural applications and in industrial bioproduction of proteins. Understanding intracellular processing of carbohydrates in this species is crucial to predicting natural carbon utilization as well as informing strategies in metabolic engineering. Here, we applied stable isotope-assisted metabolomics profiling and metabolic flux analysis to elucidate, at high resolution, the connections of the different catabolic routes for carbohydrate metabolism immediately following substrate uptake in *B. megaterium* QM B1551. We performed multiple ^13^C tracer experiments to obtain both kinetic and long-term ^13^C profiling of intracellular metabolites. In addition to the direct phosphorylation of glucose to glucose-6-phosphate (G6P) prior to oxidation to 6-phosphogluconate (6P-gluconate), the labeling data also captured glucose catabolism through the gluconate pathway involving glucose oxidation to gluconate followed by phosphorylation to 6P-gluconate. Our data further confirmed the absence of the Entner–Doudoroff pathway in *B. megaterium* and showed that subsequent catabolism of 6P-gluconate was instead through the oxidative pentose–phosphate (PP) pathway. Quantitative flux analysis of glucose-grown cells showed equal partition of consumed glucose from G6P to the Embden–Meyerhof–Parnas (EMP) pathway and from G6P to the PP pathway through 6P-gluconate. Growth on fructose alone or xylose alone was consistent with the ability of *B. megaterium* to use each substrate as a sole source of carbon. However, a detailed ^13^C mapping during simultaneous feeding of *B. megaterium* on glucose, fructose, and xylose indicated non-uniform intracellular investment of the different carbohydrate substrates. Flux of glucose-derived carbons dominated the gluconate pathway and the PP pathway, whereas carbon flux from both glucose and fructose populated the EMP pathway; there was no assimilatory flux of xylose-derived carbons. Collectively, our findings provide new quantitative insights on the contribution of the different catabolic routes involved in initiating carbohydrate catabolism in *B. megaterium* and related *Bacillus* species.

## Introduction

*Bacillus megaterium*, an aerobic bacterium ubiquitous in a diverse range of environments, has been of special investigative interest for its applications in promoting plant health (Eppinger et al., 2011; Santos et al., 2014), bioremediation of contaminants (Quinn et al., 1989), and industrial bioproduction (Vary, 1994; Vary et al., 2007; Biedendieck et al., 2010). Despite extensive genetic characterization of *B. megaterium*, detailed elucidation of its metabolic network is still lacking (Kanehisa and Goto, 2000; Eppinger et al., 2011; Kanehisa et al., 2017). Much of what is known about carbon metabolism in *Bacillus* species is derived from *Bacillus subtilis*, the primary model organism for Gram-positive bacteria (Sauer et al., 1997; Dauner and Sauer, 2001; Dauner et al., 2001; Fuhrer et al., 2005). Previous metabolic studies highlighted incongruent metabolic fluxes between *B. subtilis* and *B. megaterium* species (Sauer et al., 1997; Dauner and Sauer, 2001; Dauner et al., 2001; Fuhrer et al., 2005; Furch et al., 2007a,b; Tannler et al., 2008; Youngster et al., 2017). Metabolic flux modeling has been performed on mutant strains of *B. megaterium* that lacked specific metabolic functions (Furch et al., 2007b). Therefore, the operational network of wild-type *B. megaterium* remains to be evaluated experimentally. Of particular interest is an investigation of the different catabolic routes that initiate carbohydrate metabolism in *B. megaterium* due to the ubiquitous presence of carbohydrate-containing feedstocks.

**FIGURE 1:**
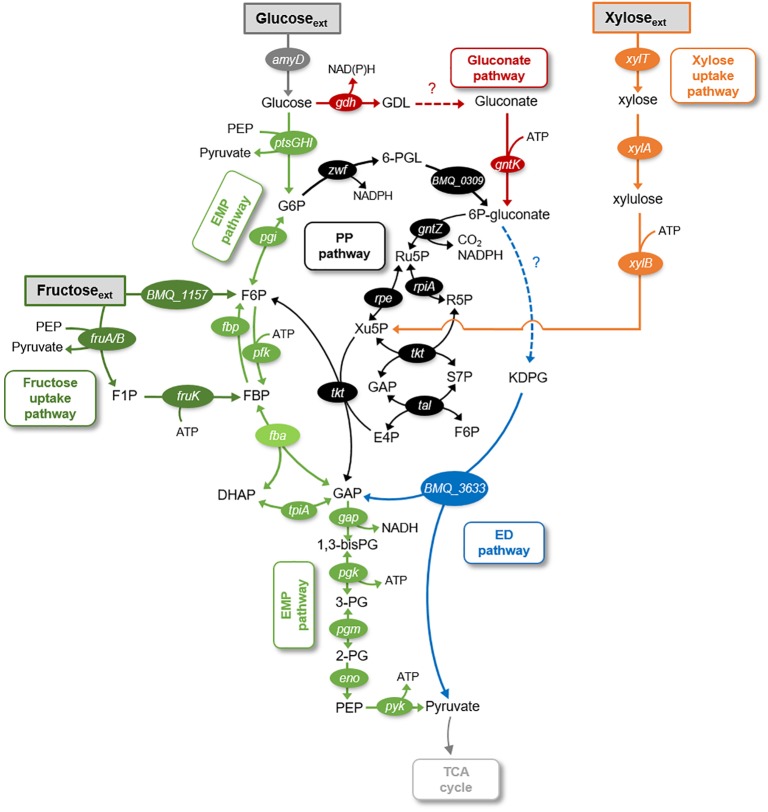
Schematic of metabolic pathways involved in glucose, fructose, and xylose catabolism in *B. megaterium* QM B1551. The following pathways are emphasized: gluconate pathway (in red), the Entner–Doudoroff (ED) pathway (in blue), the Embden–Meyerhof–Parnas (EMP) pathway (in light green), the pentose–phosphate (PP) pathway (in black), the fructose uptake pathway (in dark green), and the xylose uptake pathway (in orange). The gene annotated corresponding to each metabolic enzyme is placed next to the respective reaction arrow. The information for the network was compiled from the KEGG database (Kanehisa and Goto, 2000; Kanehisa et al., 2017) and MetaCyc database (Caspi et al., 2012). The metabolite abbreviations are as follows: GDL, glucono-1,5-lactone; G6P, glucose-6-phosphate; 6-PGL, 6-phosphogluconolactone; 6P-gluconate, 6-phosphogluconate; KDPG, 2-keto-3-deoxy-6-phosphogluconate; Ru5P, ribulose 5-phosphate; Xu5P, xylulose 5-phosphate; S7P, sedoheptulose 7-phosphate; E4P, erythrose 4-phosphate; F6P, fructose 6-phosphate; FBP, fructose 1,6-bisphosphate; DHAP, dihydroxyacetone-3-phosphate; GAP, glyceraldehyde 3-phosphate; 1,3-bisPG, 1,3-biphosphoglycerate; 3PG, 3-phosphoglycerate; 2PG, 2-phosphoglycerate; PEP, phosphoenolpyruvate. The enzymes corresponding to the gene annotations are as follows: *amyD*, carbohydrate ABC transporter permease AmyD; *ptsG*, PTS system glucose-specific transporter subunit IIBC; *ptsH*, phosphocarrier protein HPr; *ptsI*, PTS system transporter I; *gdh*, glucose 1-dehydrogenase; *pgi*, glucose-6-phosphate isomerase; *gntK*, gluconate kinase; *BMQ_1157*, fructokinase; *xylT*, xylose permease; *xylA*, D-xylose isomerase; *xylB*, xylokinase; *BMQ_0309*, 6-phosphogluconolactonase; *BMQ_3633*, 2-dehydro-3-deoxyphosphogluconate aldolase; *fruA*, PTS system fructose-specific II subunit IIA; *fruB*, PTS system fructose-specific II subunit IIB; *zwf*, glucose-6-phosphate 1-dehydrogenase; *gntZ*, phosphogluconate dehydrogenase; *rpe*, ribulose-phosphate 3-epimerase; *rpiA*, ribose 5-phosphate isomerase A; *tkt*, transketolase; *tal*, transaldolase; *pfk*, 6-phosphofructokinase; *fbp*, fructose-1,6-bisphosphatase; *fba*, fructose-1,6-bisphosphate aldolase; *tpiA*, triosephosphate isomerase; *gap*, glyceraldehyde-3-phosphate dehydrogenase; *pgk*, phosphoglycerate kinase; *pgm*, phosphoglucomutase; *eno*, enolase; *pgy*, pyruvate kinase.

Here, we investigated the metabolic fluxes underlying the catabolism of glucose, fructose, and xylose in *B*. *megaterium* QM B1551. Glucose, a monomer of the biopolymers cellulose and starch, is a common carbohydrate in environmental matrices (Koegel-Knabner, 2002). The disaccharide sucrose, a dimer with glucose and fructose, is common in plant materials and can serve as a carbon source to *B. megaterium* (Youngster et al., 2017). Xylose, a pentose monosaccharide, is a major monomer in hemicellulose, an abundant component of plant cell walls (Koegel-Knabner, 2002). The enzymes involved in the uptake pathway of all three carbohydrates (i.e., glucose, fructose, and xylose) have been annotated in the genome of *B. megaterium* (Figure 1). The metabolic pathways that can be involved in carbohydrate catabolism are the Embden–Meyerhof–Parnas (EMP) pathway, the gluconate pathway, the oxidative pentose–phosphate (PP) pathway, the non-oxidative PP pathway, and the Entner–Doudoroff (ED) pathway (Figure 1).

It is well established that *Bacillus* species undergo glycolysis *via* the EMP pathway wherein glucose is first phosphorylated to glucose-6-phosphate (G6P), then isomerized to fructose-6-phosphate (F6P) before phosphorylation to fructose-1,6-bisphosphate (FBP) (Figure 1). An important step that provides the driving force for the yields of ATP downstream of the EMP pathway is the lysis of FBP to dihydroxyacetone phosphate (DHAP) and glyceraldehyde-3-phosphate (GAP) (Figure 1; Rabinowitz et al., 2015). The metabolites in the EMP pathway can also contribute to the PP pathway (Figure 1). The oxidative phase of the PP pathway involves the oxidation of G6P to 6-phosphogluconate (6P-gluconate) followed by decarboxylation to PPs; the non-oxidative phase involves the transketolase and transaldolase reactions that combine F6P and GAP to yield intermediates in the PP pathway [xylulose-5-phosphate (Xu5P), erythrose-4-phosphate (E4P), and sedoheptulose-7-phosphate (S7P)] (Figure 1). Carbon fluxes from the EMP and PP pathways, which are eventually channeled to the tricarboxylic acid (TCA) cycle, generate reducing equivalents [NAD(P)H], energy (ATP), and metabolite precursors for biomass growth (i.e., biosynthesis of ribonucleotides for RNA and DNA, biosynthesis of amino acids for proteins) (Figure 1).

There is a lack of consensus regarding the distribution of the flux from G6P to the EMP pathway relative to the flux from G6P to the PP pathway *via* 6P-gluconate (Sauer et al., 1997; Dauner et al., 2001; Furch et al., 2007a,b; Tannler et al., 2008). Certain strains of *B. subtilis* (PRF93) were reported to utilize the PP pathway more than the direct EMP pathway (Sauer et al., 1997), whereas the opposite was reported for other *B. subtilis* strains (wild-type strain 168 and mutant strains RB50::[pRF69]_n_,) (Dauner et al., 2001; Tannler et al., 2008). In one mutant strain of *B. megaterium* (WH323), carbon starvation was shown to promote usage of the EMP pathway over the PP pathway, whereas recombinant gene induction increased activity in the PP pathway relative to the EMP pathway (Furch et al., 2007a,b). Furthermore, only a forward flux of the EMP pathway has been portrayed in previous models of *Bacillus* species, including *B. megaterium* (Sauer et al., 1997; Dauner et al., 2001; Furch et al., 2007b; Tannler et al., 2008). However, the genome of *B. megaterium* QM B1551 annotates the enzymes that can operate both forward and backward fluxes through the EMP pathway (Kanehisa and Goto, 2000; Kanehisa et al., 2017; Figure 1). The contribution of both of these fluxes, which has implications for the net thermodynamic driver of the EMP pathway, has been unexplored in previous *Bacillus* studies (Sauer et al., 1997; Dauner et al., 2001; Furch et al., 2007a,b; Tannler et al., 2008).

Regarding the gluconate pathway, which involves the oxidation of glucose to gluconate followed by phosphorylation to 6P-gluconate (Figure 1), annotation of the *B. megaterium* QM B1551 genome implied an incomplete gluconate pathway (Kanehisa and Goto, 2000; Kanehisa et al., 2017). Specifically annotated in the genome were glucose-1-dehydrogenase and gluconate kinase, which convert glucose to glucono-1,5-lactone and gluconate to 6P-gluconate, respectively (Figure 1; Kanehisa and Goto, 2000; Kanehisa et al., 2017). However, the enzyme gluconolactonase for the hydrolysis of glucono-1,5-lactone to gluconate was not present, albeit this reaction can happen spontaneously in highly alkaline conditions (Figure 1; Kanehisa and Goto, 2000; Kanehisa et al., 2017). Interestingly, despite the lack of genome annotation of gluconolactonase, evidence of the gluconate pathway was found in germinating spores of *B. megaterium* QM B1551 wherein significant gluconate evolution from glucose was detected (Otani et al., 1986; Sano et al., 1988). The connection between the gluconate pathway and the remaining network for carbohydrate catabolism in *B. megaterium* remains unclear. *Pseudomonas* species, which are well known to exhibit the gluconate pathway, rely significantly on the ED pathway to connect the gluconate pathway to downstream metabolism (del Castillo et al., 2007; Nikel et al., 2015; Sasnow et al., 2016; Wilkes et al., 2018). Due to the absence of all the relevant enzymes, a functional ED pathway in *Bacillus* species has not been considered in previous metabolic studies (Furch et al., 2007a,b; Tannler et al., 2008). Specifically, in *B. megaterium* QM B1551, genome-level characterization has annotated one of the two essential enzymes required for the ED pathway (Kanehisa and Goto, 2000; Kanehisa et al., 2017) (Figure 1). This characterization has noted the presence of 2-keto-3-deoxyphosphogluconate aldolase but the upstream enzyme, 6-phosphogluconate dehydratase, is absent from the genome of *B. megaterium* QM B1551 (Figure 1). Both the gluconate pathway and the ED pathway have not been considered in previously reported metabolic network models of *Bacillus* species (Sauer et al., 1997; Dauner et al., 2001; Furch et al., 2007a,b; Tannler et al., 2008; Korneli et al., 2012).

Previous metabolic flux analysis (MFA) of *Bacillus* species has relied on ^13^C-labeled amino acid labeling to estimate labeling at specific metabolic nodes (Sauer et al., 1997; Dauner et al., 2001; Furch et al., 2007a,b; Tannler et al., 2008; Korneli et al., 2012). However, because there are only two amino acid precursors from intermediates in the PP pathway [ribose-5-phosphate (R5P) and E4P], this method is not suitable for high-resolution determination of the carbon fluxes through the PP pathway as well as the other vicinal metabolic pathways such as the gluconate pathway and the ED pathway. Furthermore, labeling of free metabolites is needed for more accurate quantitative determination of flux distribution between the EMP pathway and the adjacent pathways. Here, we employed a ^13^C-assisted metabolomic approach using high-resolution liquid chromatography–mass spectrometry (LC–MS) to detect free metabolites involved in all four pathways. We obtained both kinetic and long-term ^13^C labeling of intracellular metabolites. We combined the cellular ^13^C mapping with growth phenotypes to perform quantitative flux analysis of carbohydrate catabolism. Our results provide new insights on the metabolic network structure and flux distribution in *B. megaterium* (strain QM B1551). These findings have broader implications regarding the optimization of *Bacillus* and related bacterial species in biotechnological applications.

## Experimental Methods

### Culturing Conditions

The *B. megaterium* QM B1551 cells were obtained from the Bacillus Genetic Stock Center (Columbus, OH, United States). Cell cultures were grown at 30°C in a G24 environmental incubator shaker (New Brunswick Scientific, Edison, NJ, United States) at 220 rpm. The growth medium, which was pH-adjusted (pH 7.0) and filter-sterilized (0.22 μm nylon; Waters Corporation, MA, United States), contained the following major salts: 18.7 mM NH_4_Cl, 0.81 mM MgSO_4_, 0.034 mM CaCl_2_. 2H_2_O, 89.4 mM K_2_HPO_4_, 56.4 mM NaH_2_PO_4_.H_2_O, 8.6 mM NaCl. The trace metal concentrations were as follows: 30 μM FeSO_4_.7H_2_O, 1.9 μM H_3_BO_3_, 0.86 μM CuSO_4_.5H_2_O, 7.7 μM ZnSO_4_.7H_2_O, 0.75 μM MnSO_4_.H_2_O, 0.26 μM NiCl_2_.6H_2_O, and 0.31 μM Na_2_MoO_4_.2H_2_O. The carbohydrate composition was 330 mM C total as glucose alone (equivalent to 55 mM or 9.91 g L^-1^ glucose), fructose, alone, xylose alone, 1:1 glucose:gluconate mixture, or equimolar glucose:fructose:xylose mixture. All chemicals listed above were analytical grade, purchased from Sigma-Aldrich (St. Louis, MO, United States) and Fisher Scientific (Pittsburg, PA, United States). For all growth conditions, the cells were transferred twice into fresh growth media to ensure that the cells were well acclimated to their nutrient growth conditions prior to experimental sampling. Cell growth (three biological replicates per condition) was monitored *via* both optical density at 600 nm (OD_600_) using an Agilent Cary UV-visible spectrophotometer (Santa Clara, CA, United States) and measuring cell dry weight (in grams, gCDW). To obtain accurate reading at OD_600_ above 0.5, cell suspensions were diluted. To determine the cell dry weight (three biological replicates), we centrifuged (9,391 *g* for 5 min at 4°C) harvested 1.5-mL culture aliquots and the retentate was frozen (-20°C) overnight prior to lyophilization using a Labconco freeze-dryer (Kansas City, MO, United States). The conversion factors of g_CDW_ L^-1^ per OD_600_ were found to be: 0.61 ± 0.08 for glucose; 0.43 ± 0.10 for glucose:fructose:xylose; and 0.53 ± 0.07 for glucose:gluconate.

### Carbohydrate Consumption

The depletion of substrate in the extracellular medium was taken to account for sugar consumption; this was confirmed by intracellular ^13^C labeling (Aristilde et al., 2015). To quantify substrate depletion by exponentially growing cells under each growth condition, 0.7 mL aliquots were harvested at different times (two independent biological replicates at each timepoint) throughout cell growth and analyzed *via* ^1^H nuclear magnetic resonance (NMR). The extracted aliquots were centrifuged for 30 min at 15,871 *g* and 4°C in filter-containing (0.22-μm pore size, nylon) microcentrifuge tubes. The filtered supernatants were frozen at -20°C until further processing. In preparation for the NMR analysis, 200 μL of the filtered samples were mixed with 60 μL of 100% D_2_O, 50 μL of 6 mM 2,2-dimethyl-2-silapentane-5-sulfonate (DSS) as an internal standard, 240 μL of 100 mM sodium bicarbonate as a pH control, and 50 μL of 10 mM sodium-azide as an antimicrobial agent. Samples were stored at 4°C until analysis (Aristilde et al., 2015). The ^1^H NMR measurements were performed using a Varian Unity INOVA 600-MHz NMR spectrometer at 25°C, with a relaxation delay of 5 s, recording of 16 scans per sample, and receiver gain of 32 dB (Aristilde et al., 2015). Substrate depletion rates (in mmol gCDW^-1^ h^-1^) were determined subsequently *via* regression analysis of carbohydrate depletion over time combined with biomass growth rate.

### Metabolite Excretion

To determine excretion rates of metabolites, 50 μL of filtered cell suspensions (three biological replicates per condition) were obtained during growth and subsequently diluted with LC–MS grade water (Fisher Scientific, Pittsburgh, PA, United States) at 1:20 v/v during early exponential phase, or 1:200 v/v during late exponential and stationary phases. Different dilution ratios were used to account for elevated concentrations of extracellular metabolites in the media as a function of cell growth. The samples were stored at 4°C (for 5 h or less) prior to processing *via* LC–MS. Excretion rates (μmol gCDW^-1^ h^-1^) were calculated *via* regression analysis.

### Kinetic Metabolite ^13^C Labeling

Kinetic flux experiments were performed to monitor *in vivo* cellular incorporation of glucose (Sasnow et al., 2016). Batch cultures (two independent biological replicates) were grown as described above for the glucose-growth condition until early exponential phase, corresponding to OD_600_ 0.4–0.6. Aliquots (3 mL) of the cultures were filtered (0.22-μm nylon filter). Each cell-containing filter disk was then placed cell-side up on an agar plate containing unlabeled carbon in minimal media to allow the cells to acclimate and reach an exponentially growing phase, corresponding to OD_600_ 0.5. To determine when this OD_600_ value was reached, cells on filters from parallel plates were rinsed off into a 3-mL suspension for OD_600_ reading. After the cells were adequately acclimated on the agar plate, the cell-containing filter disks were transferred to agar plates containing [U-^13^C_6_]-glucose and, after a set period of time following the isotopic switch (0 and 20 s; 1, 4, 12, and 30 min), the filter disks were removed from the labeled plate and metabolism was immediately quenched by flipping the disks cell-side down into a 2-mL cold (4° C) methanol:acetonitrile:water quenching solution (2:2:1). The quenched solution containing the lysed cells scraped from the filter disks was then centrifuged (5 min at 9,391 *g* and 4°C). We dried 100-μL aliquots of the supernatants under N_2_ gas prior to re-suspension in 100-μL LC–MS water for use in metabolomics analysis as described below.

### Long-Term Intracellular Metabolite ^13^C Labeling

For long-term isotopic enrichment of the intracellular metabolites, liquid cultures were prepared with the growth medium containing the major and minor salts as listed above and the following ^13^C-labeled substrates: [1,2-^13^C_6_]-glucose alone, equimolar [U-^13^C_6_]-glucose and unlabeled gluconate, or equimolar [1,2,3-^13^C_3_]-glucose, [1,6-^13^C_2_]-fructose, and unlabeled xylose. All labeled carbohydrates were purchased from Cambridge Isotopes (Tewksbury, MA, United States) or Omicron Biochemicals (South Bend, IN, United States). For these labeling experiments, batch cultures (two independent biological replicates) were grown twice in labeled minimal media and sampling was done at two timepoints (when the cells reached OD_600_ of 1.0 and 2.0) during exponential growth (Supplementary Figure S1). At each sampling time, 3 mL aliquots of cell suspensions were filtered through 0.22-μm nylon filters in a sterile environment. The cell-containing filter disks were immediately quenched in 2 mL of cold (4°C) methanol:acetonitrile:water solution (2:2:1). The lysed cells in the quenching solution were processed as described in the previous section in preparation for metabolomics analysis as described below.

### Metabolomics Analysis *via* LC–MS

The samples prepared as described above were analyzed by reversed-phase ion-pairing ultra-high performance LC (Thermo Scientific Dionex UltiMate 3000) coupled to high-resolution/accurate MS (Thermo Scientific Q Exactive quadrupole-Orbitrap hybrid MS) with electrospray ionization run in full-scan negative mode (Aristilde et al., 2017). Details of the LC–MS protocol used here were previously reported (Aristilde et al., 2017). The following metabolites were isolated by LC–MS: gluconate, 6P-gluconate, G6P, F6P, FBP, pyruvate, Xu5P, R5P, DHAP, and S7P. As previously detailed and illustrated in Aristilde, 2017, the analytical isolation of the different compound isomers (i.e., G6P/F6P, Xu5P/R5P, and DHAP/GAP) was made possible due to their chromatographic separations, despite their similar mass-over-charge ratios. Metabolite standards at various concentrations (10–1,000 nM) were also run in parallel to verify LC–MS identification and quantitation. The ^13^C labeling patterns were analyzed with the MAVEN (Metabolomic Analysis and Visualization Engine) software suite (Melamud et al., 2010; Clasquin et al., 2012). The labeling data were corrected subsequently for natural abundance of ^13^C.

### Quantitative Metabolic Flux Analysis

The MFA of *B. megaterium* cells grown on glucose alone or the glucose:fructose:xylose mixture was constrained by the following experimental data: substrate consumption rates, metabolite excretion rates, long-term ^13^C labeling data of intracellular metabolites, and biomass growth. We employed previously reported stoichiometric biomass composition for *B. subtilis* (Dauner and Sauer, 2001) to estimate metabolite effluxes (from G6P, R5P, E4P, 3-PG, pyruvate, and DHAP) to sustain the biomass requirements for cell growth under each condition. The modeling software suite 13CFLUX2 was used to conduct the MFA analysis of the pathway network involving metabolic nodes for substrate uptake, the gluconate pathway, the EMP pathway, the PP pathway, and the biomass effluxes (Weitzel et al., 2012). Metabolite secretion rates were also included for gluconate. The model was initialized using a number of free fluxes, informed by previous values of fluxes in *B. megaterium* (Furch et al., 2007a). From these free fluxes, which were unconstrained values assigned by the user to provide an initial set of flux values, the modeling algorithm optimized all flux values in accordance with the experimental constraints through an iterative process (Supplementary Tables S1, S2). At each iteration, the quality of estimated fluxes was evaluated by calculating residual errors between the model-estimated metabolite labeling data and the corresponding experimental data (Supplementary Figures S2, S3).

## Results

### Glucose Catabolism Involves the Gluconate Pathway Without the ED Pathway

**FIGURE 2:**
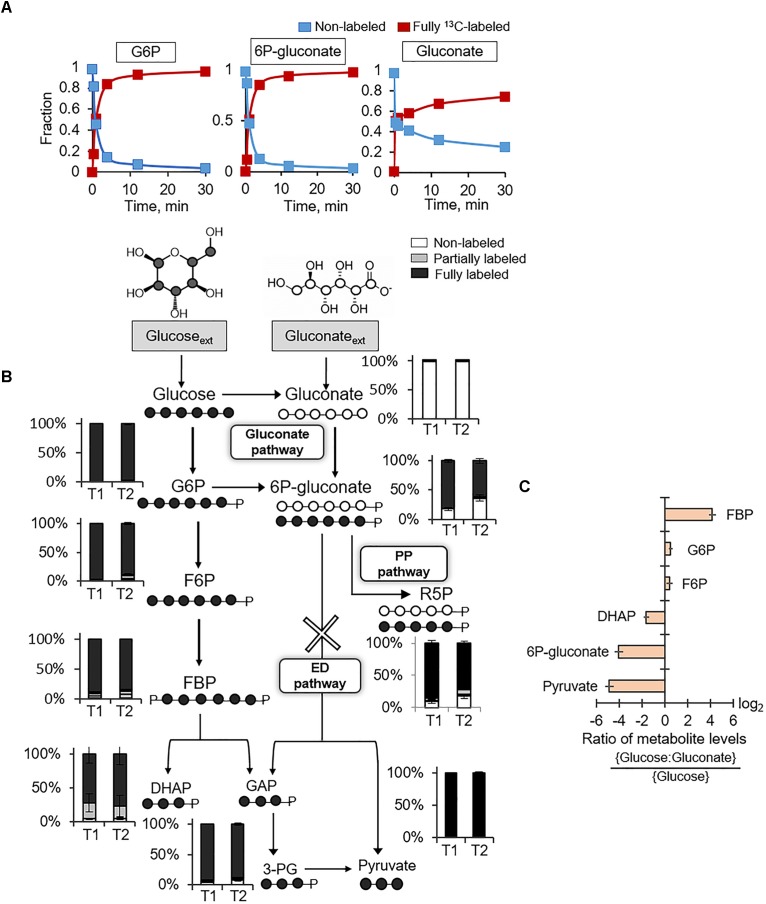
Presence of gluconate pathway and absence of Entner–Doudoroff pathway. **(A)** Kinetic ^13^C profiling of G6P, 6P-gluconate, and gluconate in cells grown on fully ^13^C-labeled glucose. **(B)** Carbon mapping and long-term intracellular ^13^C labeling at two timepoints during growth on fully ^13^C-labeled glucose and unlabeled gluconate (OD_600_ = 1, T1; OD_600_ = 2, T2). The ED pathway is marked with a cross. **(C)** Ratio of metabolite levels in cells grown on glucose alone versus cells grown on both glucose and gluconate. Measured data (average ± data range) were from two technical replicates of two independent biological replicates for each timepoint. Growth curves in Supplementary Figure S1 indicate when data were acquired during biomass growth. Definitions for metabolite abbreviations are as shown in Figure 1 caption.

Following uptake of extracellular glucose, glucose catabolism can be initiated by either phosphorylation to G6P or oxidation to gluconate (Figure 1). Kinetic ^13^C-labeling data following feeding of cells on [U-^13^C_6_]-glucose showed, by 30 min, nearly complete isotopic enrichment of G6P and 6P-gluconate and ∼75% isotopic enrichment of gluconate (Figure 2A). The ^13^C labeling of gluconate was indicative of glucose oxidation to gluconate, which was also reported in spores of *B. megaterium* QM B1551 (Otani et al., 1986; Sano et al., 1988). The plateau of gluconate ^13^C labeling below 100% may be due to carry over of residual non-labeled gluconate in the extracellular milieu because gluconate secretion was evident during growth of *B. megaterium* on glucose alone (Supplementary Table S1). Evidence of the gluconate pathway, which would include phosphorylation of gluconate to 6P-gluconate could not be resolved from the kinetic intracellular labeling from ^13^C-glucose alone. The similar labeling kinetics for 6P-gluconate and G6P implied that 6P-gluconate was primarily produced from G6P but these data could not confirm either the presence or lack of gluconate flux to 6P-gluconate (Figure 2A). To capture specifically the incorporation of gluconate-derived carbons into 6P-gluconate, we fed the cells equimolar concentrations of fully ^13^C-labeled glucose and unlabeled gluconate (Figure 2B). Across the two timepoints during exponential growth on this glucose:gluconate mixture, the fraction of non-labeled 6P-gluconate increased from 18% to 36%, confirming the presence of an active gluconate pathway (Figure 2B). However, given the high-fraction of ^13^C-labeled 6P-glcuonate (above 60%), these kinetic data also indicated that the assimilation route of glucose-derived ^13^C carbons from G6P to 6P-gluconate was favored over the gluconate pathway (Figure 2B).

There are two possible fates of 6P-gluconate: the oxidative PP pathway and the ED pathway (Figure 2B). The ED pathway involves the splitting of 6P-gluconate into GAP and pyruvate (Figure 2B). At the timepoint when 6P-gluconate had the highest non-labeled fraction (∼36%), the non-labeled fractions of both pyruvate and DHAP (an isomer of GAP) were less than 5% (i.e., within the error range of the LC–MS detection) (Figure 2B). The oxidative PP pathway was expected to generate non-labeled PPs (such as R5P) as evidence of catabolism of gluconate-derived carbons *via* this pathway (Figure 2B). We found that nearly 20% of R5P was non-labeled, implying that about 72% of the R5P pool was synthesized from 6P-gluconate *via* the oxidative PP pathway (Figure 2B). Therefore, these data collectively demonstrated that the fate of 6P-gluconate was through the oxidative PP pathway and that the ED pathway was inactive in *B. megaterium* QM B1551.

We compared the metabolite pools in cells grown on the equimolar glucose:gluconate mixture to those in cells grown on glucose alone – the total substrate carbon concentration (330 mM C) was the same in both conditions (Figure 2C). There was no appreciable change in the levels of G6P and F6P (Figure 2C). However, during growth on the glucose:gluconate mixture, the level of 6P-gluconate was severely depleted (16-fold reduction), in agreement with limited flux from gluconate to 6P-gluconate, as indicated by the labeling data (Figures 2B,C). In accordance with the lack of the ED pathway, we obtained a decrease in both DHAP (∼3-fold reduction) and pyruvate (∼30-fold reduction) during feeding on the glucose:gluconate mixture (Figure 2C). Interestingly, despite the depletion in DHAP, which is downstream of FBP, there was an accumulation in FBP (∼18-fold increase) during feeding on the glucose:gluconate mixture (Figure 2C). Given the constant level of F6P upstream of FBP, these data implied that, when gluconate is present with glucose, the flux of FBP production from F6P exceeded the flux of FBP lysis to generate DHAP and GAP (Figure 2C).

### Glucose Catabolism to Pyruvate Is Through a Partially Reversible EMP Pathway

**FIGURE 3:**
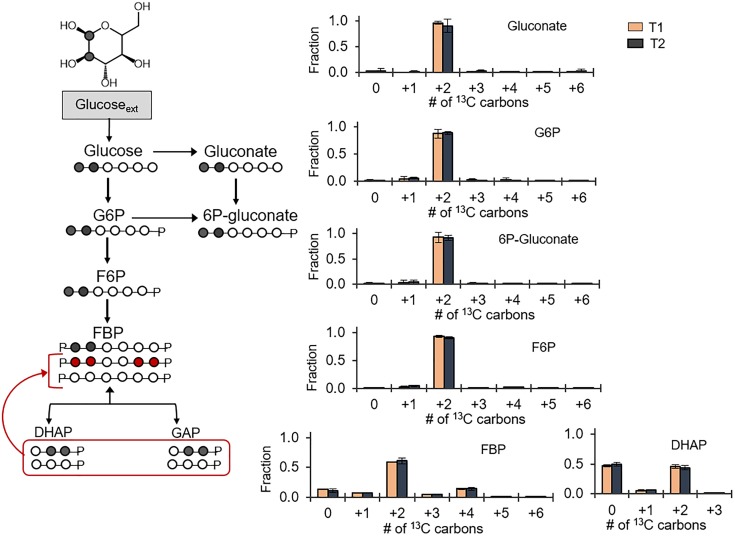
Partial reversibility of the Embden–Meyerhof–Parnas pathway. Carbon mapping (left) and metabolite labeling (right) in the gluconate pathway and the EMP pathway following feeding on [1,2-^13^C_2_]-glucose. The red colored arrows and carbon skeletons are to illustrate the formation of metabolites from backward flux in the upper EMP pathway. Measured data (average ± data range) were collected at two different timepoints during cell growth (T1, OD_600_ = 1.0; T2, OD_600_ = 2.0) from two technical replicates of two independent biological replicates at each timepoint. Growth curves in Supplementary Figure S1 indicate when data were acquired during biomass growth. Definitions for metabolite abbreviations are as shown in Figure 1 caption.

Following the ^13^C probing approach described above, [1,2-^13^C_2_]-glucose was chosen as a substrate to evaluate possible backward fluxes in the EMP pathway (Figure 3). These backward fluxes, which are implied from the annotated genome of *B. megaterium* (Kanehisa and Goto, 2000; Kanehisa et al., 2017), have not been probed previously. The forward EMP pathway involves carbon flux from glucose through G6P, F6P, FBP, and onward to DHAP and GAP following FBP lysis (Figure 3). In agreement with the assimilation of the doubly ^13^C-labeled glucose, we obtained ∼90% doubly ^13^C-labeled fractions for gluconate, 6P-gluconate, G6P, and F6P (Figure 3). However, there was a discrepancy between the labeling patterns of F6P and those of FBP (Figure 3). Compared to F6P, there was a 30% depletion in the doubly ^13^C-labeled fraction of FBP (Figure 3). Moreover, non-labeled FBP was 10%, compared to 1% in F6P; the quadruply ^13^C-labeled fraction was 14% in FBP, compared to 2% in F6P (Figure 3). Non-labeled intermediates in the EMP pathway were generated during lysis of the doubly [1,2-^13^C_2_]-FBP into [2,3-^13^C_2_] DHAP and non-labeled GAP (Figure 3). Equilibrium isotopic labeling between the isomers DHAP and GAP led to the measurement of near equal fraction (∼50%) of non-labeled and doubly ^13^C-labeled fractions in DHAP (Figure 3). Operating the EMP pathway in the reverse direction by combining the labeling scheme for DHAP and GAP to produce FBP explained the non-labeled and quadruply ^13^C-labeled fraction in FBP, in addition to the doubly ^13^C-labeled fraction (Figure 3). However, the fact that F6P was primarily doubly ^13^C-labeled indicated that the EMP pathway was only partially reversible (Figure 3).

### Glucose Catabolism and Gluconate Pathway Are Both Connected to a Primarily Oxidative PP Pathway

**FIGURE 4:**
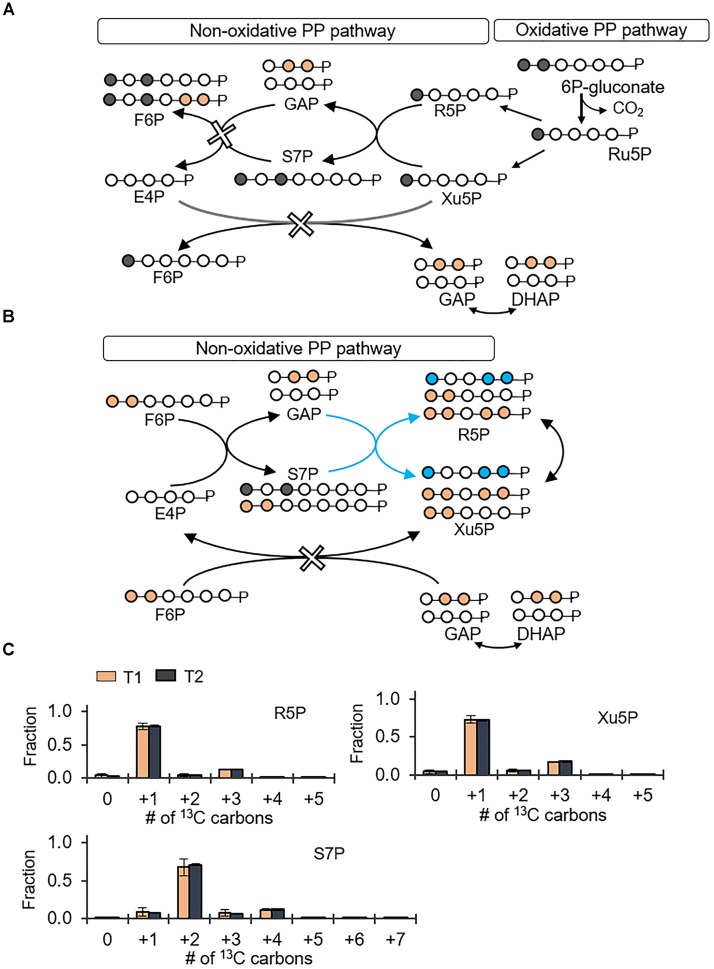
Predominance of the oxidative pentose phosphate (PP) pathway over the non-oxidative PP pathway. Carbon mapping **(A,B)** and metabolite labeling **(C)** in the PP pathway following feeding on [1,2-^13^C_2_]-glucose. Carbon mapping is shown for carbon flow from the oxidative PP pathway **(A)** or from the metabolites in the EMP pathway incorporated into the non-oxidative PP pathway **(B)**. Blue-colored circles and arrows indicate metabolite labeling patterns derived from the EMP pathway operating in the direction of the non-oxidative PP pathway. Measured data (average ± data range) were collected at two different timepoints (T1, OD_600_ = 1.0; T2, OD_600_ = 2.0) from two technical replicates of two independent biological replicates. Growth curves in Supplementary Figure S1 indicate when data were acquired during biomass growth. Definitions for metabolite abbreviations are as shown in Figure 1 caption.

The PP pathway possesses both an oxidative route and a non-oxidative route (Figures 4A,B). The oxidative PP pathway involves the decarboxylation of 6P-gluconate to Ru5P, which subsequently interconverts to R5P and Xu5P; the non-oxidative PP pathway combines metabolites from the EMP pathway, F6P and GAP, to generate eventually Xu5P and R5P (Figures 4A,B). According to our labeling scheme, decarboxylation of doubly ^13^C-labeled 6P-gluconate through the oxidative PP pathway would produce singly ^13^C-labeled R5P and Xu5P but the non-oxidative PP pathway would use EMP-pathway metabolites to generate triply ^13^C-labeled R5P and Xu5P (Figures 4A,B). We found that the metabolite labeling patterns of R5P and Xu5P were primarily singly ^13^C-labeled (77 and 72%, respectively) and only up to 18% of these metabolites were triply ^13^C-labeled (Figure 4); in accordance with the labeling of Xu5P and R5P, we also obtained primarily doubly ^13^C-labeled S7P (Figure 4). Thus, the labeling patterns of Xu5P and R5P were consistent with a significant involvement of the oxidative PP pathway with relatively minor contribution from the non-oxidative pathway (Figure 4). Therefore, through the oxidative PP pathway, PP metabolites were primarily produced from 6P-gluconate, which was generated from both the gluconate pathway and the direct glucose catabolism through G6P.

### Simultaneous Processing of Different Carbohydrates Is Partitioned Into Different Catabolic Routes

**FIGURE 5:**
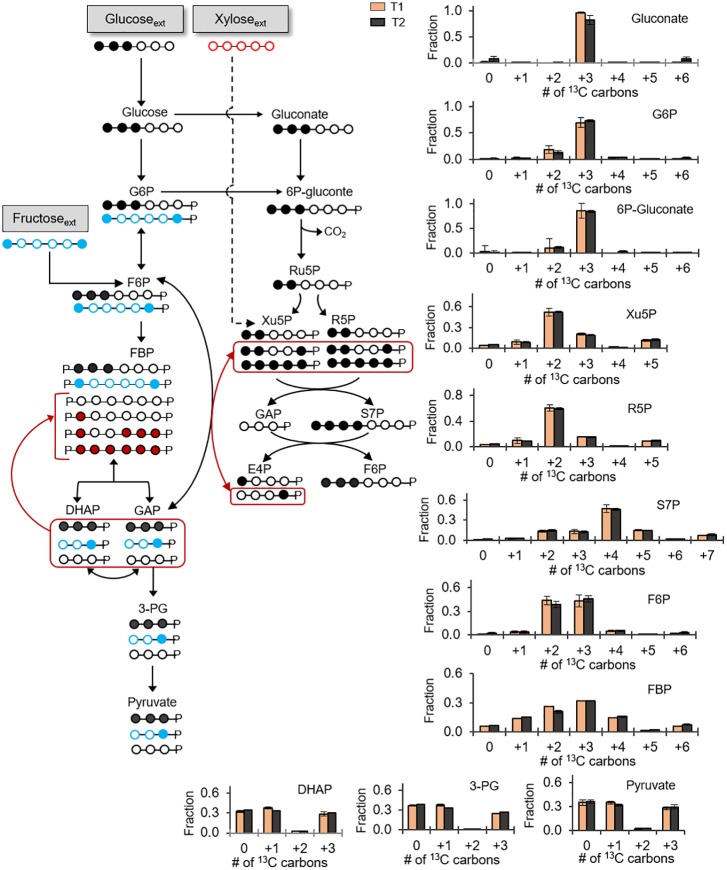
Non-uniform incorporation of carbohydrate mixture into metabolism. Carbon mapping (left) and metabolite labeling (right) following feeding on [1,2,3-^13^C_3_]-glucose (black circles), [1,6-^13^C_2_]-fructose (blue circles), and unlabeled xylose (red circles). The red-colored arrows and carbon skeletons are to illustrate the formation of metabolites that are a result of the partially reversible Embden–Meyerhof–Parnas (EMP) pathway or backward flux from EMP pathway to the non-oxidative pentose–phosphate pathway. Measured data (average ± data range) were collected at two different timepoints during cell growth (T1, OD_600_ = 1.0; T2, OD_600_ = 2.0). Growth curves in Supplementary Figure S1 indicate when data were acquired during biomass growth. Definitions for metabolite abbreviations are as shown in Figure 1 caption.

Previous reports of carbon metabolism and metabolic flux modeling have focused on glucose-grown *Bacillus* species (Sauer et al., 1997; Dauner et al., 2001; Tannler et al., 2008). However, glucose is often present with other carbohydrates in carbon feedstocks (Wendisch et al., 2016). In addition to glucose, we were able to obtain growth of the *B. megaterium* cells when the sole carbon source was given as fructose (another common hexose) or xylose (a common pentose) (Supplementary Figure S1). These data thus indicated that, in accordance with the genome annotation (Figure 1), the cells have the transporters and catabolic pathways to assimilate these carbohydrates. We also investigated how *B. megaterium* QM B1551 processes simultaneously glucose, fructose, and xylose by monitoring intracellular incorporation of equimolar carbon-equivalent concentrations of [1,2,3-^13^C_3_]-glucose, [1,6-^13^C_2_]-fructose, and unlabeled xylose (Figure 5). The labeling data revealed that a non-uniform intracellular routing of each substrate in the metabolic network (Figure 5).

Consistent with the oxidation of the triply ^13^C-labeled glucose, gluconate was predominantly triply ^13^C-labeled (at ∼85%) (Figure 5). From the phosphorylation of triply ^13^C-labeled glucose to G6P, the G6P pool had a high triply ^13^C-labeled fraction (60–73%) with a lesser amount of doubly ^13^C-labeled (19%) fraction (Figure 5). Downstream of G6P and gluconate, 6P-gluconate was ∼85% triply ^13^C-labeled and 11% doubly ^13^C-labeled (Figure 5). The enrichment in triply ^13^C-labeled fraction (by up to 20%) from G6P to 6P-gluconate emphasized the contribution of gluconate to generate 6P-gluconate, thus underscoring an active gluconate pathway in initiating the catabolism of glucose-derived carbons during growth on multiple carbohydrates (Figure 5). The small fraction of doubly ^13^C-labeled fractions in both G6P and 6P-gluconate reflected the minor incorporation of fructose-derived carbons in these metabolites (Figure 5).

Notably, for the F6P pool, we obtained an equal amount (∼45% each) of triply ^13^C-labeled fraction and doubly ^13^C-labeled fractions, respectively from glucose-derived and fructose-derived carbons (Figure 5). Forward flux through the EMP pathway was expected to generate ^13^C labeling for FBP similar to F6P and, subsequently, the splitting of the triply ^13^C-labeled and doubly ^13^C-labeled fractions of FBP would generate equal proportions of non-labeled, singly ^13^C-labeled, and triply ^13^C-labeled fractions of triose-phosphates (Figure 5). In accordance with this labeling scheme in the EMP pathway, we did obtain an equal proportion of non-labeled, singly ^13^C-labeled and triply ^13^C-labeled fractions of DHAP, 3-PG, and pyruvate (Figure 5). However, in addition to doubly and triply ^13^C-labeled FBP (at 20–32%), there were other ^13^C-labeled fractions in FBP (at 5-15%) (Figure 5). Specifically, the latter fractions were non-labeled, singly ^13^C-labeled, quadruply ^13^C-labeled, and sextuply ^13^C-labeled fractions of FBP (Figure 5). Thus, the labeling pattern of FBP reflected backward flux from the combination of DHAP and GAP to produce FBP (Figure 5). However, this backward flux was not extended to F6P (Figure 5). Therefore, the partial reversibility of carbon flux in the upper EMP pathway previously discussed with the glucose-grown cells was also evident in cells grown on the glucose:fructose:xylose mixture (Figures 3, 5). It is important to note that, in addition to indicating that the upper EMP pathway was only partially reversible, the distinct difference between the labeling patterns of F6P and FBP revealed that F6P was the entry point for the catabolism of fructose-derived carbons (Figure 5).

With respect to xylose assimilation, the incorporation of xylose-derived carbons was expected to introduce non-labeled carbons in the PP pathway. However, non-labeled fractions in the PP pathway metabolites (R5P, Xu5P, and S7P) were only 5% or less (Figure 5). By contrast, in accordance with the oxidative PP pathway, triply ^13^C-labeled 6P-gluconate from assimilated glucose-derived carbons led to 52–60% doubly ^13^C-labeled fractions in both R5P and Xu5P (Figure 5). Consistent with the combination of these latter two metabolites to produce S7P, we obtained quadruply ^13^C-labeled S7P (∼46%) (Figure 5). Despite the predominance of the oxidative PP pathway, minor fractions of other ^13^C isotopologs of the PP pathway metabolites were consistent with minor flux of the non-oxidative PP pathway (Figure 5). In sum, during growth on the carbohydrate mixture, glucose-derived carbons primarily populated the gluconate pathway and the oxidative PP pathway, both fructose-derived and glucose-derived carbons equally contributed to the EMP pathway, and the assimilation of xylose was insignificant (Figure 5).

### Quantitative Flux Modeling During Growth on Glucose Alone Versus a Mixture With Glucose, Fructose, and Xylose

**FIGURE 6:**
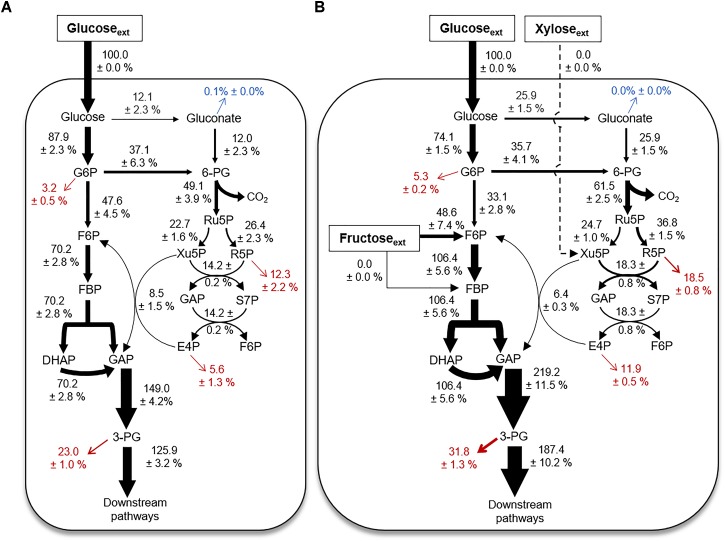
Cellular metabolic flux distributions. Metabolic flux analysis of *B. megaterium* QM B1551 during feeding on **(A)** [1,2-^13^C_2_]-glucose or **(B)** a mixture of [1,2,3-^13^C_3_]-glucose, [1,6-^13^C_2_]-fructose, and unlabeled xylose. All fluxes were normalized to 100% glucose uptake and the thickness of each arrow was scaled to this flux percentage. Fluxes toward biomass production are shown in red. Gluconate excretion is shown in blue. Flux values (average ± data range) were generated using experimental data from two independent biological replicates, at OD_600_ = 2. Growth curves in Supplementary Figure S1 indicate when data were acquired during biomass growth. Definitions for metabolite abbreviations are as shown in Figure 1 caption.

Using the ^13^C labeling data and the growth phenotypes, we performed MFA to quantitate explicitly up to 18 fluxes through the different catabolic routes for carbohydrate utilization in *B. megaterium* during growth on glucose alone or with fructose and xylose (Figure 6). The fluxes were normalized to the glucose uptake rate in both growth conditions (Figure 6) – the absolute fluxes are presented in Supplementary Table S2. The cellular fluxes demonstrated that different catabolic routes were accentuated in *B. megaterium* depending on whether the cells were processing a single or a mixture of carbohydrate substrates (Figure 6).

Compared to the metabolism of glucose alone, there was a two-fold increase of the flux through the gluconate pathway in cells grown on the mixture (Figures 6A,B). This increased flux through the gluconate pathway during processing of the mixture was accompanied by a decrease (by up to 12%) in the carbon flux from G6P to F6P (Figures 6A,B). The increased carbon flux through the gluconate pathway led to a 17% increase in the activity of the oxidative PP pathway, from 6P-gluconate to Ru5P, in cells grown on the mixture relative to glucose alone (Figures 6A,B). Subsequent fluxes through the PP pathway were also increased (Figures 6A,B). The exception was the increase (by 25%) in the reaction flux that combines Xu5P and E4P to produce F6P and GAP (Figures 6A,B), which may be influenced by the influx of fructose-derived carbons into the EMP pathway (Figures 5, 6A,B). During growth on the mixture, the uptake flux from fructose to F6P was about half of the glucose uptake flux (Figure 6B). The second annotated pathway for fructose uptake (i.e., from fructose to FBP), was found to be inactive (Figures 1, 6B). Due to the incorporation of fructose-derived carbons at the metabolic node of F6P, the flux through the remaining EMP pathway downstream of F6P, was increased by up to 70% (Figures 6A,B). Thus, the overall flux profile in the gluconate pathway, EMP pathway, and PP pathway was reprogrammed to accommodate the assimilation of fructose (Figures 6A,B).

## Discussion

Here, we employed ^13^C-carbon mapping of intracellular metabolism to address knowledge gaps regarding the routes for carbohydrate catabolism in *B. megaterium* QM B1551. We also resolved inconsistencies from previous studies on *Bacillus* species regarding the relative flux from glucose catabolism through either the EMP pathway or the PP pathway (Sauer et al., 1997; Dauner et al., 2001; Furch et al., 2007a,b; Tannler et al., 2008; Korneli et al., 2012). Our ^13^C labeling data presented evidence of both glucose oxidation to gluconate and flux from gluconate to 6P-gluconate, thus confirming the presence of the complete gluconate pathway in *B. megaterium* (Figures 2A,B). Our data also revealed that flux through the gluconate pathway was compromised by congestion at the gluconate node (Figures 2A,B). Further studies are needed to elucidate the regulatory mechanism for the gluconate pathway in *B. megaterium* QM B1551, which was beyond the scope of our data. Interestingly, in *Pseudomonas* species, gluconate pathway is strongly linked to a very active ED pathway (Nikel et al., 2015; Sasnow et al., 2016; Wilkes et al., 2018). Although the ED pathway was widely neglected in prior MFAs conducted on *Bacillus* species (Furch et al., 2007a,b; Tannler et al., 2008), genomic characterization of *B. megaterium* QM B1551 annotated one of the two essential enzymes in the ED pathway (Figure 1; Kanehisa and Goto, 2000; Kanehisa et al., 2017). However, our data demonstrated that the ED pathway was not active in *B. megaterium* QM B1551 (Figure 2B). Regarding the traditional glycolytic pathway (i.e., the EMP pathway), our flux modeling determined a net forward flux through the EMP pathway (Figure 6), in agreement with previous MFA studies on *Bacillus* species including *B. megaterium* (Sauer et al., 1997; Dauner et al., 2001; Furch et al., 2007a,b; Tannler et al., 2008). However, our discrete metabolite labeling data revealed that the FBP lysis was reversible (Figures 3, 5). The consequence of this reversibility regarding the optimization of bioproduct generation in *B. megaterium* species remains to be determined.

The resolution of metabolite labeling in the PP pathway of *Bacillus* species has previously been deduced from ^13^C labeling of their amino acid derivatives (Sauer et al., 1997; Dauner et al., 2001; Furch et al., 2007b; Korneli et al., 2012). Here, using a LC–MS approach to obtain the labeling patterns of three free metabolites specific to the PP pathway, we determined that the flux through the oxidative PP pathway (i.e., from 6P-gluconate to Ru5P) was ∼6-fold to ∼10-fold greater than the flux through the non-oxidative PP pathway (from F6P and GAP) (Figures 4C, 6B). This relative contribution of the two routes in the PP pathway was larger than previous published estimates, which reported a 3.5- to 5-fold greater activity of the oxidative PP pathway activity than non-oxidative PP pathway in *Bacillus* species (Sauer et al., 1997; Dauner et al., 2001; Furch et al., 2007b). The increased flux through the oxidative PP pathway determined from our modeling fluxes implied that the *B. megaterium* QM B1551 cells can afford a greater production of reducing power through the generation of NADPH than previously estimated.

While glucose is the substrate of choice to elucidate the metabolism of most bacterial model systems, a mixture of carbohydrates is typically present in environmental systems or in engineered bioreactors for industrial applications (Wendisch et al., 2016). Biomass growth on only glucose, fructose, or mannose indicated that *B. megaterium* can rely on each substrate as a sole source of carbon in its metabolism (Supplementary Figure S1). However, our metabolic study of cells fed on equimolar carbon-equivalent concentrations of glucose, fructose, and xylose revealed that *B. megaterium* QM B1551 utilizes different catabolic routes to process this carbohydrate mixture (Figures 5, 6). No appreciable incorporation of xylose-derived carbons was detected (Figure 5). Carbons derived from fructose and glucose contributed equally to the flux in the EMP pathway from F6P to the triose phosphates (Figure 5). The gluconate pathway was only populated by glucose-derived carbons (Figure 5). Flux of glucose-derived carbons was also significant from the gluconate pathway through the oxidative PP pathway (Figures 5, 6). Our findings thus demonstrated that *B. megaterium* QM B1551 grown on a mixture can exhibit both complete repression of a given carbohydrate assimilation and preferential channeling of the assimilated carbohydrates into different catabolic routes. These findings have important implications for the processing of different carbon feedstocks. Follow-up metabolic flux studies and proteomics profiling are needed to gain further insights on how *B. megaterium* regulates this hierarchy in carbohydrate catabolism in response to different composition and concentration of carbohydrate mixtures.

Given the emergent usage of different feedstocks in engineered bioproduction, it is critical to understand the metabolic network in biotechnologically important bacterial species in response to various carbon sources (Wendisch et al., 2016). Due to the abundance of carbohydrate-containing feedstocks, we have focused here on determining the flux through the different catabolic pathways available for carbohydrate processing in *B. megaterium* QM B1551. The ^13^C-metabolomics approaches employed here present a powerful tool to elucidate new pathways and gain insights on existing pathways involved in the intracellular network of cellular metabolism. These approaches could be instrumental to the broader field of metabolic characterization of environmentally-relevant bacteria and optimization of bacterial cells in biotechnological applications.

## Author Contributions

LA supervised the research. JW and LA designed the research, conducted data analysis, and wrote the manuscript. JW and TY performed the experiments. JW and CM performed the quantitative flux modeling. All authors contributed to the final draft of the manuscript.

## Conflict of Interest Statement

The authors declare that the research was conducted in the absence of any commercial or financial relationships that could be construed as a potential conflict of interest.
