# Supplementary material for: Flux Connections Between Gluconate Pathway, Glycolysis, and Pentose–Phosphate Pathway During Carbohydrate Metabolism in Bacillus megaterium QM B1551
Source: Front Microbiol. 2018 Nov 21;9:2789. doi: 10.3389/fmicb.2018.02789 (PMC6262346; doi:10.3389/fmicb.2018.02789)
Supplement: Supplementary file 1 [file Data_Sheet_1.PDF]

Journal: Frontiers in Microbiology
Research Topic: Fluxomics and Metabolic Flux Analysis in Systems Biology

Flux Connections between Gluconate Pathway, Glycolysis, and Pentose-Phosphate Pathway during Carbohydrate Metabolism in *Bacillus Megaterium* QMB1551

Julie A. Wushensky,[1] Tracy Youngster,[2] Caroll M. Mendonca,[1] Ludmilla Aristilde[1,2,*]

[1]*Department of Biological and Environmental Engineering, College of Agriculture and Life Sciences, Cornell University, Ithaca, NY 14853, USA*
[2]*Soil and Crop Sciences Section, School of Integrative Plant Science, College of Agriculture and Life Sciences, Cornell University, Ithaca, NY 14853, USA*

*Corresponding Author:
Phone: (607) 255-6845. Fax: (607) 255-4449. E-mail: ludmilla@cornell.edu

SUPPLEMENTARY MATERIAL

Fig. S1
Table S1
Table S2
Fig. S2
Fig. S3

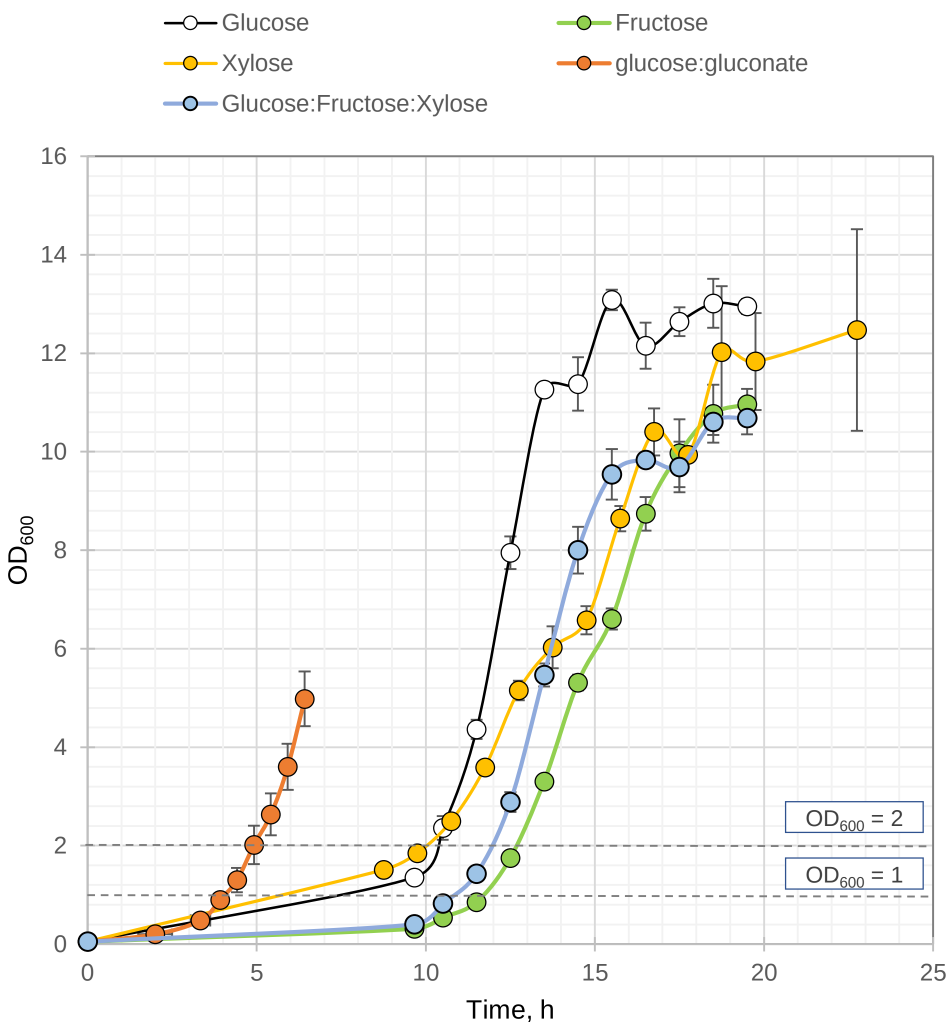

Fig. S1. Growth curves of *B. megaterium* QM B1551 grown on 330 mM total carbon of glucose alone (white circles), fructose alone (light green circles), xylose alone (yellow circles), glucose:gluconate mixture (orange circles), and equimolar mixture of glucose, fructose, and xylose (light blue circles). Data (average ± standard deviation) were from three independent biological replicates.

Table S1. Growth parameters[a] of *B. megaterium* QM B1551.

**Growth Condition[b]**	Glucose	Glucose:Fructose:Xylose
**Growth Rate (hr^{-1})**	0.71 ± 0.04	0.57 ± 0.07
**Uptake Rate (mmol $gCDW^{-1}$ hr^{-1})**	5.30 ± 1.61[c]	Glucose: 0.28 ± 0.06[c]
		Fructose: 0.13 ± 0.05
		Xylose: negligible
**Gluconate Excretion rate (µmol $gCDW^{-1}$ hr^{-1})**	4.37 ± 1.76	2.30 ± 0.81

[a]The values in the table represent means ± standard deviation. Data were calculated from three independent biological replicates (*n* = 3).
[b]The total caron-equivalent concentration of the growth substrates was 330 mM C.
[c]Uptake rate of glucose into the periplasm.

Table S2. Intracellular metabolic rates determined from quantitative flux modeling of the metabolism of [1,2-$^{13}C_2$]-glucose in *B. megaterium* QM B1551 using the 13CFLUX2 software. These metabolic fluxes are illustrated in Fig. 7 in the main text, where they have been normalized to glucose uptake. Refer to the legends of Fig. 1 in the main text for the abbreviations of the metabolite names.

**Reactions**	**[1,2-$^{13}C_2$]-glucose**	**[1,2,3-$^{13}C_3$]-glucose, [1,6-$^{13}C_2$]-fructose, unlabeled xylose**
	mmol gCDW^{-1} h^{-1}	*mmol gCDW^{-1} h^{-1}*
$Gluc_{ext}$ -> $Gluc_{peri}$	5.30 ± 0.00	0.28 ± 0.00
$Gluc_{peri}$ -> G6P	4.66 ± 0.09	0.21 ± 0.00
$Gluc_{peri}$ -> $Glucn_{peri}$	0.64 ± 0.09	0.07 ± 0.00
$Glucn_{peri}$-> 6P-Glucn	0.64 ± 0.09	0.07 ± 0.00
G6P -> 6P-Glucn	1.97 ± 0.24	0.10 ± 0.01
GAP -> 3-PG	7.89 ± 0.16	0.61 ± 0.02
3-PG -> downstream processes	6.67 ± 0.12	0.52 ± 0.01
DHAP -> GAP	3.72 ± 0.11	0.30 ± 0.01
FBP -> DHAP + GAP	3.72 ± 0.11	0.30 ± 0.01
F6P -> FBP	3.72 ± 0.11	0.30 ± 0.01
G6P -> F6P	2.52 ± 0.17	0.09 ± 0.00
6P-Glucn -> Ru5P	2.60 ± 0.15	0.17 ± 0.00
Ru5P-> R5P	1.40 ± 0.09	0.10 ± 0.00
Ru5P -> Xu5P	1.20 ± 0.06	0.07 ± 0.00
Xu5P + R5P -> GAP + S7P	0.75 ± 0.01	0.05 ± 0.00
S7P + GAP -> E4P + F6P	0.75 ± 0.01	0.05 ± 0.00
E4P + Xu5P -> F6P + GAP	0.45 ± 0.05	0.02 ± 0.00
$Fruc_{ext}$ -> F6P	--	0.14 ± 0.01
$Fruc_{ext}$ -> FBP	--	0.00 ± 0.00
Xyl_{ext} -> Xyl	--	0.00 ± 0.00
E4P -> Biomass	0.30 ± 0.05	0.03 ± 0.00
G6P -> Biomass	0.17 ± 0.02	0.01 ± 0.00
3PG -> Biomass	1.22 ± 0.04	0.09 ± 0.00
R5P -> Biomass	0.65 ± 0.08	0.05 ± 0.00
$Glucn_{in}$ -> $Glucn_{ext}$	0.1 ± 0.00	0.00 ± 0.00

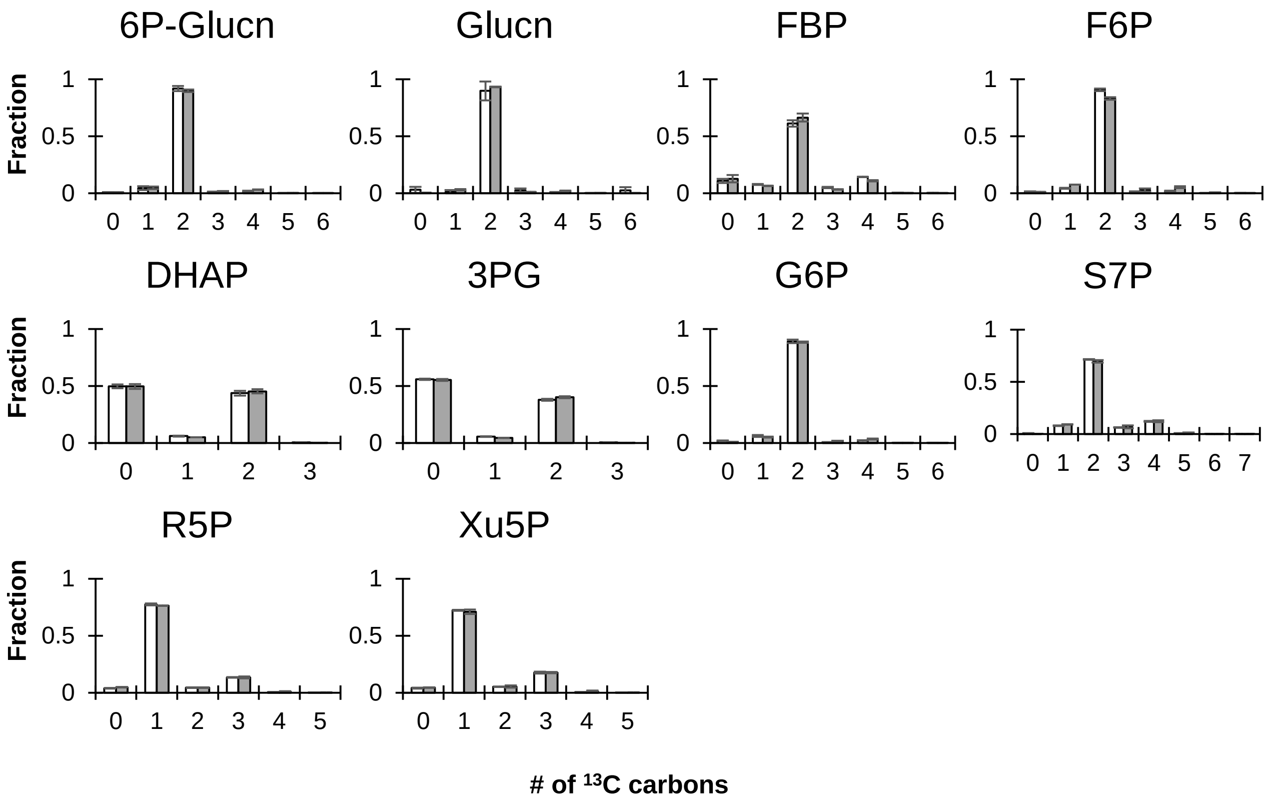

Fig. S2. Experimentally-determined (white bars) and model-estimated (grey bars) isotopomer distributions in the metabolite labeling patterns during *B. megaterium* QM B1551 growth on [1,2-$^{13}C_2$]-glucose. Data (average ± standard deviation) were from optimizations of experimental data obtained from two independent biological replicates.

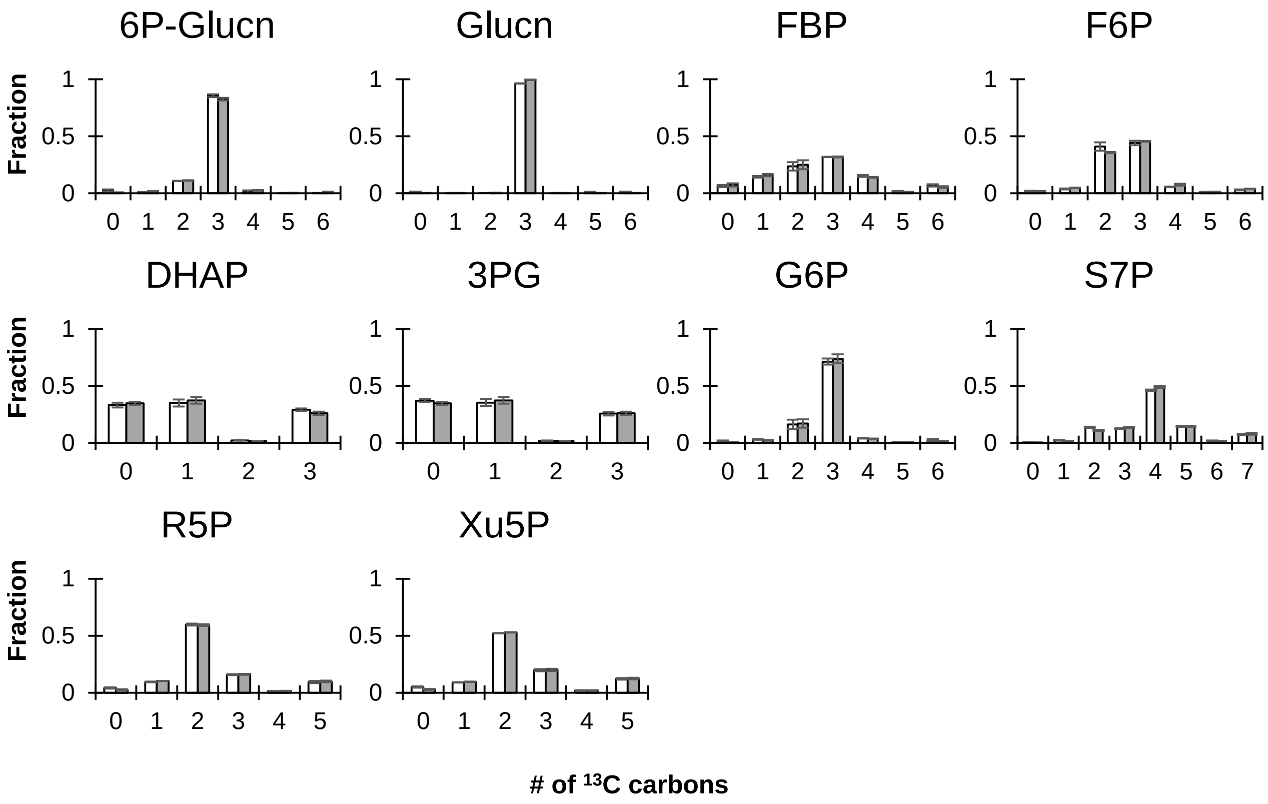

Fig. S3. Experimentally-determined (white bars) and model-estimated (grey bars) isotopomer distributions in the metabolite labeling patterns during *B. megaterium* QM growth on a mixture of [1,2,3-$^{13}C_3$]-glucose, [1,6-$^{13}C_2$]-fructose, and unlabeled xylose. Data (average ± standard deviation) were from optimizations of experimental data obtained from two independent biological replicates.
